# An Algorithmic Approach to the Management of Shoulder Instability

**DOI:** 10.5435/JAAOSGlobal-D-19-00168

**Published:** 2019-12-23

**Authors:** Alex E. White, Nirav K. Patel, Christopher J. Hadley, Christopher C. Dodson

**Affiliations:** From the Sidney Kimmel Medical College at Thomas Jefferson University, Philadelphia, PA (Mr. White); VCU Health, Medical College of Virginia at Virginia Commonwealth University, Richmond, VA (Dr. Patel); and The Rothman Orthopaedic Institute at Thomas Jefferson University, Philadelphia, PA (Mr. Hadley and Dr. Dodson).

## Abstract

The recurrence of anterior shoulder instability can be as high as 86.7% in high-risk patients who are treated nonoperatively after their first incident of instability. CT and MR arthrography are necessary for preoperative imaging and assessment of glenoid bone loss. Patient expectations in conjunction with appropriate preoperative imaging are critical for surgical planning. Arthroscopic shoulder stabilization is often sufficient in most cases where glenoid bone loss is minimal, with recurrent dislocation rates close to 4% in the literature. Open stabilization procedures are generally indicated in patients with greater than 20% glenoid bone loss.

Traumatic shoulder instability is a common clinical entity in sports medicine, with a reported incidence of 1.7% in the general cohort.^1^ Some of the most salient risk factors for recurrent shoulder instability include younger age, male sex, and participation in collision sports.^2–4^ Recurrence of primary shoulder dislocations has been reported to be between 38% and 80% in the literature when managed nonoperatively.^5,6,7,8,9,10,11^ In one particular cohort study, young men recurred at the highest rate, 86.7% of the time.^3^ Nonoperative management has traditionally been advocated as initial treatment in all cases of shoulder instability. However, this idea has lost support because research has shown persistently high rates of recurrent shoulder instability with nonoperative management, particularly in people younger than 25 years.^12^ Surgery for primary dislocation has been shown to decrease the incidence of recurrent instability markedly in this same cohort.^13^ A number of patient-specific factors should be considered in each case; however, surgical management has become the mainstay of treatment for both primary and recurrent shoulder instability. Despite this relative consensus in favor of operative management, indications for the various surgical techniques continue to exhibit great heterogeneity from one surgeon to the next.

The surgical management of shoulder instability is not straight-forward. There are numerous patient-oriented and anatomic factors to be considered. Patient-oriented factors include age, level of activity, and the nature of sport participation (overhead sport, contact level, etc). The essential anatomic lesions of recurrent instability are (1) Bankart lesion (including Perthes, anterior labroligamentous periosteal sleeve avulsion [ALPSA], and glenolabral articular disruption lesions), (2) capsular injury and laxity (including humeral avulsion of glenohumeral ligament lesion), (3) Hill-Sachs lesion, and (4) glenoid dysplasia. Of these essential lesions, the size of glenoid bone defect (most commonly a bony Bankart lesion) has been most clearly associated with a high risk of recurrent instability. Studies have shown that a notable risk of glenohumeral instability occurs at close to 21% glenoid bone loss.^14^ Thus, much debate has emerged around the management of shoulders with notable glenoid bone loss. The spectrum of surgical techniques done for anterior shoulder stabilization is vast, but some of the common procedures today include the arthroscopic Bankart repair, Bankart plus remplissage, double row capsulolabral repair, Latarjet technique, and glenoid open reduction with internal fixation (ORIF).

In this review, we will discuss the epidemiology of shoulder instability and propose a surgical treatment algorithm including consideration for the more common surgical procedures including arthroscopic surgery with a standard labral repair, arthroscopy with remplissage, double row repair, and indications for open procedures such as a Bankart repair with or without a concomitant humeral sided capsular shift and modified Latarjet. This algorithm, as supported by the literature, will add to previously existing knowledge on the indications for surgery in anterior shoulder dislocation with special attention paid to the varying degree of glenoid bone loss.

## Clinical Evaluation

A full history and physical examination are essential components in the evaluation of a shoulder dislocation. With the former, it is important to gather how the injury occurred, how the shoulder was reduced, how many times the shoulder has dislocated in the past, the patient's goals, and the patient's age at first dislocation. Understanding this information sets the stage for the remainder of the clinical, radiographic, and surgical course of the patient. Provencher et al^15^ listed the following characteristics as risk factors for glenoid bone loss: high-energy mechanism of injury, arm abduction and extension at the time of initial dislocation, most instability occurring at midrange of motion (20° to 60° of abduction), instability during normal activities of daily living, and a long history of instability. In addition, care should be taken to rule out other shoulder pathology, such as fracture, rotator cuff tendinopathy, glenohumeral arthritis, and biceps tendinitis, particularly in patients older than 40 years. Finally, it is important to consider the timing of a physical examination relative to the time of injury. An examination in the acute setting can be confused by global pain and inflammation, and thus, allowing a patient to recover for a few days before examination may enhance its accuracy.

The principles of an appropriate physical examination include an assessment of the amount of passive glenohumeral translation and provocative testing to reproduce the symptoms and/or apprehensions of the patient. In addition, assessment of generalized patient laxity is important for determining etiology and planning management. Traditional scoring systems such as the Beighton score have shown poor reliability in this assessment, and thus, the use of more specific shoulder joint laxity measures such as external rotation, abduction, and sulcus sign should continue to be used preferentially.^16^ The load and shift test should be used to evaluate anterior translation in three positions of glenohumeral elevation in the plane of the scapula: neutral (arm at side), 45°, and 90°. All of these should be done with the patient in mild external rotation. Posterior translation should also be assessed with the load and shift test, where the patient is placed in mild internal rotation and varying degrees of abduction. A gross shoulder deformity, positive shoulder apprehension in the midrange of abduction (30° to 90°) with limited external rotation, and reproducible anterior translation of the humeral head over the glenoid rim are all suggestive of glenoid bone loss.^15^ The combined anterior apprehension and relocation tests are additional physical examination maneuvers useful for identifying patients with shoulder instability. Examination findings combined with a detailed patient history provide a clinical context for subsequent diagnostic imaging.

## Diagnostic Imaging

Imaging for shoulder dislocation should begin with plain radiographs. These are important in assessing for gross lesions and to rule out associated fractures. An internal rotation or Grashey view^17^ can best visualize the presence of a Hill-Sachs lesion. Bony Bankart lesions are best seen with an AP view. The position of the humeral head in the glenoid cavity is best visualized with an axillary view. Anterior glenoid rim defects and Hill-Sachs lesions are best seen on Bernageau, Stryker Notch, and west point views.^18^ Bony defects on plain radiograph are most likely to be seen in chronic, recurrently unstable shoulders, and thus, plain radiograph is generally poor for assessing bone loss in the shoulder.

MRI is the most useful modality for evaluating the soft tissues of the shoulder joint. In patients younger than 40 years, an MRI or MR arthrogram (MRA) is an important test to identify a Bankart and other labral lesions. MRA has been shown to be more sensitive and specific for panlabral lesions at 88% sensitivity and 93% specificity compared with 76% and 87% for MRI, respectively.^19^ However, in the same study, MRI was found to be more sensitive and specific for anterior labrum lesions, whereas MRA had better sensitivity and specificity for SLAP lesions.^19^ For patients older than 40 years, MRI has additional utility for excluding rotator cuff tears and assessing labral pathology. Other lesions, such as the Perthes lesion, can be well-visualized with an MRA.

**Figure 1:**
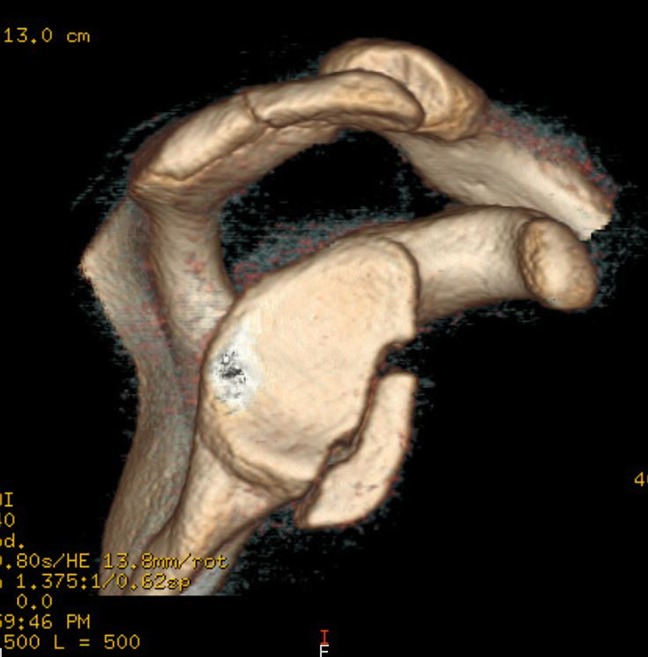
Three-dimensional reconstruction CT image with “en face” sagittal view, which can assess for glenoid dysplasia, bony Bankart lesions, glenoid bone loss, and Hill-Sachs lesions.

CT plays an important role in patients who are suspected for notable glenoid bone loss or other bony lesions. In particular, the “en face” sagittal view with 3D reconstruction can assess for glenoid dysplasia, bony Bankart lesions, and glenoid bone loss. In particular, gaining a quantitative understanding of the percentage of glenoid bone loss is best done with the “en face” sagittal three-dimensional CT (Figure 1) and is an important factor in preoperative planning. A cadaveric study showed that both CT and three-dimensional CT are superior than MRI and radiographs in quantifying glenoid bone loss.^20^ Thus, in patients with the requisite history and examination findings suggestive of notable glenoid bone loss, a CT or 3D reconstructed CT should be attained before surgery.

There are two primary concepts in the literature that seek to explain the biomechanical aspects of shoulder instability with bone loss and their implications for surgical management. The first of these concepts addresses engaging and nonengaging Hill-Sachs lesions. A Hill-Sachs lesion involves a cortical defect in the posterolateral aspect of the humeral head. Those lesions that engage do so with a corresponding Bankart or anterior inferior glenoid lesion and are traditionally contraindicated for arthroscopic repair. A newer concept of on-track and off-track lesions, however, has gained favor for assessing glenohumeral instability with bone loss. In on-track lesions, the Hill-Sachs lesion remains within the glenoid's normal zone of contact in the end range of motion. In off-track lesions, however, the edge of the Hill-Sachs lesion translates more medially than the medial border of the glenoid's normal zone of contact.^21^ This newer on-track and off-track concept has been shown to reliably predict surgical outcomes and recurrent instability.^22^

## Treatment

### Arthroscopic Versus Open Bankart Repair

The open Bankart repair was once regarded as the benchmark procedure for anterior shoulder instability. Despite a shift toward arthroscopic stabilization procedures, the open Bankart repair remains a viable option with the appropriate indications. More recently, the addition of a concomitant capsular shift for capsular tightening has gained popularity. Studies comparing open Bankart repair with inferior capsular shift to arthroscopic Bankart repair continue to show a lower incidence of recurrent instability in the open procedure. The arthroscopic procedure, however, generally has the benefit of fewer postoperative range of motion limitations.^23^ There is no agreed on threshold for bone loss because it pertains to open versus arthroscopic surgery; however, as surgeons become more comfortable with arthroscopic procedures, there has been and will continue to be a natural decline in the number of open procedures.^24^

**Figure 2:**
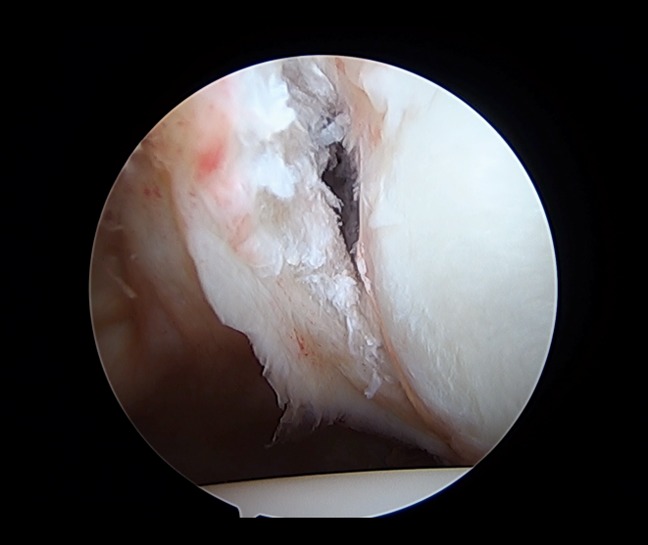
Arthroscopic image showing an anterior labral tear after elevation from the glenoid rim, consistent with a Bankart lesion and minimal bone loss.

**Figure 3:**
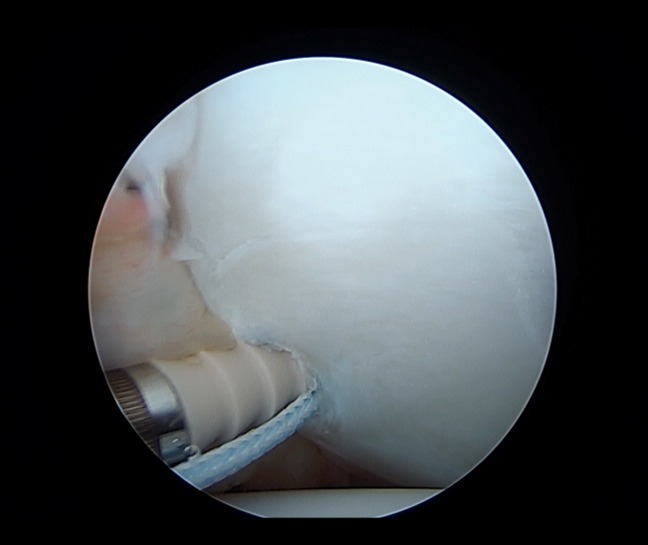
Arthroscopic image showing displacement of the first glenoid anchor placed during an arthroscopic Bankart repair. Pilot hole was drilled before anchor placement.

**Figure 4:**
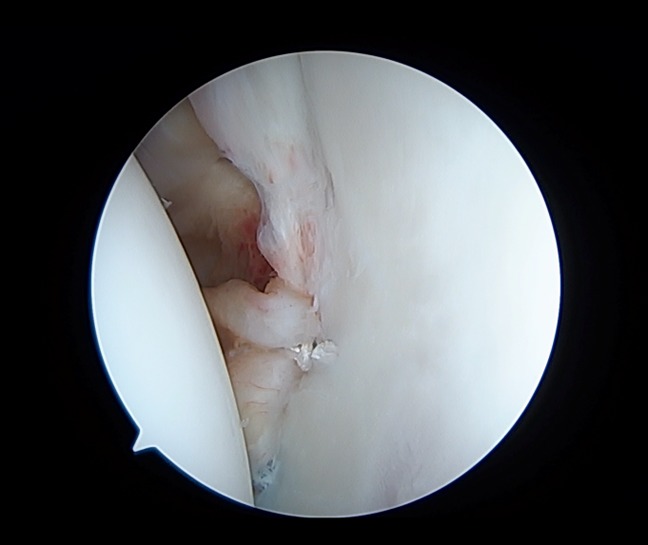
Final construct of arthroscopic Bankart repair with anchors used to reduce and stabilize the anterior labrum back to its anatomic position on the glenoid.

The arthroscopic Bankart repair is a minimally invasive surgical approach in the management of anterior shoulder dislocation. The general goal of this procedure is to repair an anterior labral tear by reattaching it to the glenoid rim with suture anchors. The standard arthroscopic positioning is used with either the beach chair or lateral decubitus position. Initial anatomic landmarks should be identified and marked, notably the borders of the acromion, coracoid process, acromioclavicular joint, and distal clavicle. The posterior viewing portal is established roughly 2 cm distal and 1 cm medial to the posterolateral border of the acromion. A diagnostic arthroscopy should always be done to identify relevant pathology before beginning the procedure (Figure 2). A low anteroinferior portal within the deltopectoral interval should be created using needle localization to enter the joint just above the subscapularis tendon. An anterosuperior portal is then created distal to the anterosuperior border of the acromion, again using needle localization to create a portal lateral to the biceps tendon in the rotator interval. Once identified, the Bankart lesion is aggressively mobilized with an elevator, and the donor glenoid area is then débrided and decorticated with a shaver. The mobility of the capsulolabral lesion should be assessed, and the muscle belly of the subscapularis should be visualized. Finally, fixation of the Bankart lesion is done (Figures 3 and 4), ideally using a minimum of four suture anchors.^25,26^ Notable modifications for the arthroscopic Bankart procedure include a variety differing fixation methods, including simple suture, suture anchor with horizontal mattress suture, double-loaded suture anchor with simple suture, and knotless suture anchors.^27^ In addition, a double row repair of the capsulolabral complex to the glenoid or a posterior, inferior capsular plication with suture anchors may be used in certain circumstances as an arthroscopic “plus” procedure.^28^

Outcomes for arthroscopic Bankart repair are mixed (Table 1). A systematic review of the literature reports recurrent dislocation rates to range between 6.3% and 35.3%.^36^ Numerous risk factors are believed to play a role in recurrent instability in these patients, thus explaining the widely variable rates reported. Some of these factors include age <20 years, male sex, greater number of dislocations, higher level of sport participation, fewer number of surgical anchors used, greater portion of glenoid bone loss, presence of a Hill-Sachs or ALPSA lesion, and generalized ligamentous laxity. One particular study that did not exclude patients based on glenoid bone loss criteria experience a recurrent instability rate of 18% after arthroscopic Bankart repair with suture anchors.^37^ A second study by Burkhart and DeBeer,^38^ however, separated the results of arthroscopic Bankart repair between patients with notable glenoid bone less and those without any glenoid bone loss at all. Those without any preoperative glenoid bone loss recurred at just 4%, whereas those with notable bone defects, as defined as an inverted pear glenoid or engaging Hill-Sachs lesion, recurred at a rate of 67%.^38^ These findings suggest that arthroscopic Bankart repair is most appropriate for patients without notable glenoid bone loss.

**Table 1:** Clinical Outcomes of Arthroscopic Bankart Repair

Study	Total No. of Patients in Study	Mean Age (Range)	Complications	Dislocation Events (%)	Rowe Score (Mean)
Kim et al^29^	32	38 (30–62)	0	6.3	90
Castagna et al^30^	31	26.3 (17–46)	0	19.4	80.1
Franceschi et al^31^	60	27.6 (15–40)	0	8.3	88
Van der Linde et al^32^	68	31	0	35.3	NR
Boughebri et al^33^	45	29.4 (17–58)	0	8.9	82.6
Plath et al^34^	100	27.7 (16–57)	0	21.0	NR

NR = not reported

### Bankart Repair and Remplissage

The goal of the Bankart repair and remplissage procedures done concomitantly is to restore the articular surface of the glenoid while simultaneously eliminating an engaging Hill-Sachs lesion. Both procedures can be successfully done arthroscopically. The main goal of the remplissage procedure is to fill the humeral head Hill-Sachs lesion using tissue from the rotator cuff. To do this technique, the remplissage component must be completed before labral fixation. In general, a single suture anchor is placed in to the center of the Hill-Sachs lesion, and the sutures are retrieved through the posterior/superior rotator cuff, which is then tied down to fill the defect on the humeral head.

In a recent study, investigators done the Bankart repair and remplissage on a patient cohort with an average glenoid bone loss of 5.4%.^39^ The overall recurrent dislocation rate was 11.8%; however, the average glenoid bone loss in these patients was 14%.^39^ Patients returned to their previous intensity and level of sport at a rate of 81%, and average time to return to sports was 7 months.^39^ Boileau et al^40^ further showed success with this procedure specifically in patients with minimal glenoid bone loss. Thus, indications for the arthroscopic Bankart repair plus remplissage procedure include patients with less than 25% bone loss and off-track Hill-Sachs lesions.^41^

### Double Row Capsulolabral Repair

The goal of the double row capsulolabral repair is to create a stronger, more stable reattachment of the anterior capsulolabral complex. Conceptually, this technique achieves a greater surface area for fixation by using both the anterior glenoid rim and the anterior glenoid neck, thereby restoring the original capsulolabral footprint. The procedure uses suture anchors for the double row fixation, where the first fixation row is placed medially on the glenoid neck with two suture anchors, and the second fixation row is placed laterally on the glenoid rim with three suture anchor.^42^ In most cases, this entire procedure should be done arthroscopically.

Reported clinical outcomes for the double row capsulolabral repair are sparse. Two studies, however, have reported favorable outcomes without any intraoperative or postoperative complications and successful return to sport.^42,43^ Additional studies are needed to assess the long-term, reproducible success of this procedure. This procedure should be done on patients who have less than 20% glenoid bone loss and good-quality capsulolabral tissue.

### Latarjet Technique

The Latarjet technique aims to create a “triple blocking effect” to restore stability to the glenohumeral joint. The first step of the procedure comprises drilling the coracoid for a single screw to be used for fixation before osteotomy. The horizontal limb of the coracoid process is then sectioned between insertions of the coracobrachialis and the pectoralis minor using a chisel. Next, a subscapularis tenotomy is done to allow shortening of the tendon during closure, or a longitudinal approach parallel to the muscle fibers can be used to spare the subscapularis tendon. The scapula neck is then cleared, and the coracoid is laid flat with the posterior surface against the glenoid neck, which is then fixed in place outside the joint using single screw.

Finally, the subscapularis and capsule are repaired by suture over bone graft. Since the classic technique was introduced, there have been several modifications to the procedure.^44^ For instance, a number of studies have differed in their coracoid osteotomy site, subscapularis approach, coracoid fixation site, orientation of the coracoid graft, method of fixation, and the type of capsular repair.^44^

The Latarjet procedure has had good reported success in the literature (Table 2). This is predicated on the “triple blocking effect,” wherein the conjoined tendon does a sling-like action in shoulder abduction and external rotation, the coracoid osteotomy creates an anterior bone block, and the capsular repair provides a bumper effect. The nature of the procedure, wherein a coracoid bone graft is translocated to the anterior glenoid, makes it a suitable procedure in patients with notable glenoid bone loss. Studies have reported recurrent dislocation rates between 0% and 9.6%.^36^ The authors in one study in particular operated on patients with greater than 25% glenoid bone loss and experienced just 4.7% recurrent instability at a mean of 52 months postoperatively.^56^ Surgical complications, however, remain a hindrance for the Latarjet procedure because one study in particular reported a complication rate of 25%, with nerve injury and infection being the most prominent problems.^48^

**Table 2:** Clinical Outcomes of Latarjet Technique

Study	Total No. of Patients	Mean Age (Range) (Years)	Complications	Dislocation Events (%)	Rowe Score (Mean)
Singer et al.^45^	14	25 (18–36)	0	0.0	85.3
Allain et al^46^	58	27.5 (15–58)	16	0.0	86.6
Hovelius et al^47^	118	27 (15–57)	21	3.4	89.4
Schroder et al^48^	52	20.5 (18–22)	6	9.6	81.8
Hovelius et al^49^	97	27.8 (17–51)	3	5.2	87.5
Neyton et al^50^	37	23.4 (17–33)	5	0.0	93
Hovelius et al^51^	34	26 (17–40)	0	8.8	NR
Laderman et al^52^	117	28.4 (16–55)	11	0.9	NR
Bouju et al^53^	58	26.7 (NR)	19	1.7	NR
Mizuno et al^54^	68	29.4 (16–58)	5	2.9	89.6
Gordins et al^55^	31	26.7 (15–39)	6	3.2	NR

NR = not reported

### Glenoid Open Reduction and Internal Fixation

The glenoid ORIF is a procedure that is specifically indicated in the setting of notable glenoid fracture displacement. Rockwood and Green postulated that ORIF is best indicated in cases of greater than 20% glenoid bone loss with chronic instability and fracture displacement.^57^ The procedure uses a standard deltopectoral approach, and a vertical incision of the inferior portion of the subscapularis muscle and capsule is made approximately 1 centimeter from its humeral insertion. A 1.25-mm threaded Kirschner wire is used to reduce the glenoid fragment, and a second threaded Kirschner wire is drilled to fix the fragment to the glenoid body. A third Kirschner wire is drilled to guide a threaded 3.5-mm cannulated screw for fixation, and then, the screw position is generally checked under fluoroscopy. The capsule and subscapularis muscle are then reattached with sutures.^37^

In general, patients recover well from glenoid ORIF with good outcomes. One study of 20 patients noted no occurrence of redislocation at a median follow-up of 3.8 years.^37^ Patients had a median Rowe score of 90, with two patients experiencing malpositioned screws that led to erosion of the humeral head. This study, however, consisted of patients with types 1a and 2 glenoid rim fractures only, and 21% of patients experienced disappointing functional outcomes.^37^ There are a limited number of studies with reported outcomes for this procedure, and thus, it should be reserved for cases of obvious and severely displaced glenoid fractures of greater than 20% bone loss.

## Algorithmic Approach and Recommendations

**Figure 5:**
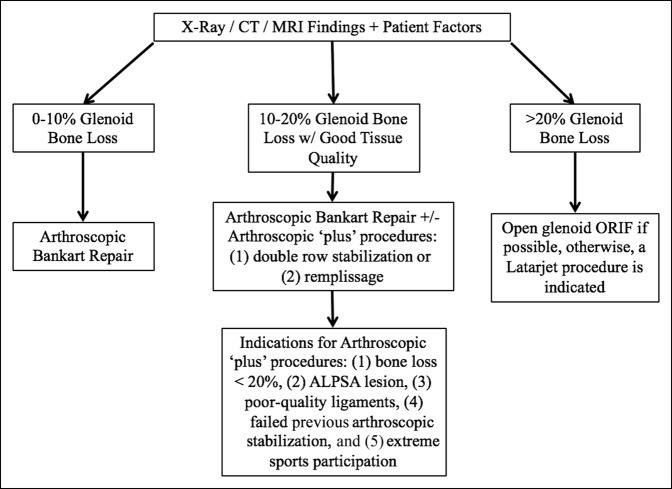
Treatment algorithm for anterior shoulder instability. ALPSA = anterior labroligamentous periosteal sleeve avulsion, ORIF = open reduction with internal fixation

Glenohumeral joint stability encompasses a combination of dynamic and static joint stabilizers. To provide optimal treatment, good preoperative imaging is critical. Through the combination of our clinical experience and a comprehensive evaluation of the literature, we recommend a treatment algorithm based primarily on the degree of glenoid bone loss and patient expectations (Figure 5). Arthroscopic stabilization remains the mainstay of treatment for most uncomplicated cases of shoulder instability and can provide good to excellent results. There is a subset of cases, however, that are at especially high risk of recurrence and should be considered for advanced stabilization techniques.

First, patient expectations, age, and lifestyle should play a prominent role in the decision-making process. In general, if glenoid bone loss is between 0% and 10%, we consider the patient to be a good candidate for an arthroscopic Bankart repair. Spiegl et al^58^ recommended nonoperative treatment in patients with osseous Bankart lesions less than 5%; however, we would consider operative management in cases where the patient is young and expects to return to an active lifestyle—particularly if returning to contact sports. It is important to consider all risk factors associated with recurrent instability, such as young age, male sex, and participation in contact sports when making the decision for surgical management.^2–4^ In addition, athletes who require preserved external rotation of the shoulder for optimal athletic performance should be considered for arthroscopic stabilization instead of open procedures if at all possible.

Patients with glenoid bone loss between 10% and 20%, who have good tissue quality, are generally indicated for an arthroscopic anterior stabilization with “plus” procedures, such as the double row stabilization or remplissage, as indicated. Some of the specific indications for this include ALPSA lesions, engaging Hill-Sachs lesion, poor-quality ligaments, a previous failed arthroscopic stabilization, and extreme sports participation—as these factors all predispose patients to failure of isolated arthroscopic Bankart repair.^1^ The remplissage procedure, in particular, has shown promising results in the case of an engaging Hill-Sachs lesion.^59^ External rotation, however, is sometimes compromised in this procedure as a result of the mechanical blocking from the posterior capsule fixation in the Hill-Sachs defect.^59,60^ This loss of motion is a consideration in the patient expectations and should be weighed with the risks and benefits of other more or less invasive procedures. Arthroscopic double row capuslolabral stabilization can be done in an attempt to recreate the normal anatomic capsulolabral footprint in patients who have 10% to 20% glenoid bone loss, without a notable Hill-Sachs defect but good tissue quality. There are limited data to support the clinical efficacy of this procedure, but it should remain an option for the appropriately trained surgeon.

Finally, in glenoid bone defects of greater than 20% bone loss, we recommend an ORIF if possible. In instances where ORIF is not possible, we suggest an open Latarjet procedure to create the previously described “triple blocking effect.” At this point in time, arthroscopic procedures remain inadequate for these patients. Unfortunately, the results reported for ORIF in anterior shoulder stabilization remain suboptimal, and the procedure imparts the risk of possible screw malalignment. The results from the Latarjet procedure have been promising when primary glenoid ORIF is not possible; however, it carries notable risks that prohibit us from recommending it as first line in these cases.

## Conclusion

In summary, arthroscopic stabilization provides good to excellent results in most cases of anterior shoulder instability. There is a subset of patients, however, with notable osseous lesions that remain at particularly high risk of recurrence and require open procedures for adequate stabilization. The literature has shown that anterior shoulder instability with less than 20% of glenoid bone loss can be treated arthroscopically in one form or another. For the subset of patients with 10% to 20% glenoid bone loss, however, there may be indication for supplemental or arthroscopic “plus,” procedures such as the remplissage in the cases of an engaging Hill-Sachs lesion or double row capsulolabral repair in an attempt to recreate the normal anatomic labral insertion. For those with greater than 20% glenoid bone loss, however, there is an absolute indication for open stabilization—particularly with ORIF when possible and then open Latarjet procedure otherwise. Additional research is necessary to validate our recommendations. In particular, additional high-level studies are needed to evaluate the efficacy of double row capsulolabral repairs in cases of glenoid bone loss. In addition, efforts should be made to limit the complications associated with the Latarjet procedure if we hope to provide it as a viable primary surgical option in cases of notable glenoid bone loss.
